# Dataset of *Nematostella vectensis* Hsp70 isoform interactomes upon heat shock

**DOI:** 10.1016/j.dib.2019.104580

**Published:** 2019-09-28

**Authors:** Laura E. Knighton, Nitika, Donald Wolfgeher, Adam M. Reitzel, Andrew W. Truman

**Affiliations:** a Department of Biological Sciences, The University of North Carolina at Charlotte, Charlotte, USA; b Department of Molecular Genetics and Cell Biology, The University of Chicago, Chicago, USA

**Keywords:** Molecular chaperones, Hsp70, Interactomes, Nematostella, Vectensis, Client proteins, Heat shock

## Abstract

*Nematostella vectensis* is an estuarine sea anemone that has emerged as a model species to characterize molecular responses to physiological stressors due to its exposure to diverse, extreme abiotic conditions. In marine cnidarians, Hsp70 proteins can be effective biomarkers to determine mechanisms of physiological acclimation and evolutionary adaptations to environmental stress: a pressing issue as concerns about climate change grow. Here we show the results of affinity purification mass spectrometry of three *Nematostella vectensis* Hsp70 isoforms, NvHsp70A, B and D when expressed in untreated and heat shocked yeast cells lacking their native Hsp70s. We identified a total of 1031 interactors for the three NvHsp70 isoforms, 549 or which were shared. NvHsp70 isoform interactions altered substantially under heat stress with 17% of NvHsp70A, 51% of NvHsp70B and 20% of NvHsp70D interactions increasing after exposure to 39 °C for 2 hours. For further interpretation of the data presented in this article, please see the research article “Dynamic remodeling of the interactomes of *Nematostella vectensis* Hsp70 isoforms under heat shock**”**.

**Table:** Specifications Table

Subject	Molecular biology
Specific subject area	*Proteomics, protein interactions*
Type of data	*Mass spectrometry data*
How data were acquired	Mass spectrometry. Thermo LTQ Orbitrap XL
Data format	*.Raw
Parameters for data collection	Heat shock-induced interactome of NvHsp70 isoforms purified from yeast
Description of data collection	Yeast cells lacking ssa1-4 and expressing either HIS_6_-tagged NvHSP70A, B or D were untreated or stressed at 39 °C for 2 hours. HIS_6_-NvHsp70 complexes were purified by IMAC and processed by mass spectrometry.
Data source location	The University of Chicago, Chicago, IL, USA
Data accessibility	Data are available with this article. In addition the mass spectrometry proteomics data have been deposited to the ProteomeXchange Consortium via the PRIDE (https://www.ebi.ac.uk/pride/archive/) partner repository with the dataset identifier PXD012144
Related research article	Knighton LE^1^, Nitika^1^, Waller SJ^1^, Strom O^1^, Wolfgeher D^2^, Reitzel AM^3^, Truman AW^4^. Dynamic remodeling of the interactomes of *Nematostella vectensis* Hsp70 isoforms under heat shock**”** Journal of Proteomics 10.1016/j.jprot.2019.103416.

**Table:** 

**Value of the Data** • These data demonstrate that highly similar Hsp70 isoforms bind unique sets of client and co-chaperone proteins. • Chaperone researchers may use this data to identify interesting client proteins that may have human homologs involved in disease. • The methodologies and analyses presented here will inform researchers on how to best characterize chaperone interactomes from non-model organisms.

## 1 Data

To determine differences in NvHsp70 isoform interactions, we expressed HIS- tagged NvHsp70A, B and D isoforms in yeast, then purified their complexes from cells exposed to either 30 °C or 39 °C for 2 hours. These complexes were digested and were analyzed by mass spectrometry. Our analysis identified 1171 interactors associated with the three NvHsp70 isoforms. Out of the total number of interactors, 549 (47%) were found to interact with all three of the isoforms. NvHsp70B interacted with the most proteins, 1031 (88%) in total, of which 211 (18%) were unique to this isoform. NvHsp70A interacted with a total of 891 (76%), of which 81 (7%) were unique. Lastly, NvHsp70D interacted with a total of 639 (55%), of which 38 (3%) were unique to the D isoform. NvHsp70A and NvHsp70B shared 240 interactors (21%), whereas NvHsp70A and NvHsp70D only shared 21 interactors (1.8%) and NvHsp70B and NvHsp70D 31 interactors [1].

## 2 Experimental design, materials, and methods

Yeast cultures were grown in YPD (1% yeast extract, 2% peptone, 2% glucose) or in SD (0.67% yeast nitrogen base, 2% glucose) supplemented with the appropriate nutrients to select for plasmids and gene replacements. NvHsp70A, B, and D were amplified from cDNA synthesized from RNA isolated from *Nematostella vectensis* adults originally collected from Sippewissett, MA and inserted into the pAG415GPD-ccdB vector for expression in yeast using In-Fusion cloning as in Ref. [2]. All inserts were sequence confirmed with Sanger sequencing. For HIS-tagged versions of NvHsp70A, B, and D, the Hsp70 isoforms were PCR amplified in a similar manner to the untagged versions except using a Forward primer that contained an N-terminal 6xHIS tag and GG linker (primer sequence available on request). The resulting PCR product was cloned via In-Fusion cloning into pAG415GPD-ccdB. Each NvHsp70 isoform construct was transformed into yeast strain ssa1–4Δ [3] using PEG/lithium acetate. After restreaking onto media lacking leucine, transformants were streaked again onto media lacking leucine and containing 5-fluoro-orotic acid (5-FOA), resulting in yeast that expressed NvHsp70 isoforms as the sole cytoplasmic Hsp70 in the cell.

### 2.1 Purification of NvHsp70 interactomes from yeast

Yeast clones expressing each NvHsp70 isoform were grown to an OD_600_ of 0.5 in YPD media. Cells were then incubated at 39 °C for two hours (heat shock) or incubated at 30 °C (untreated). His-tagged proteins were purified as follows: 200μg of protein extract was incubated with His-Tag Dynabeads (Invitrogen) at 4 °C for 20 minutes with gentle agitation. Dynabeads were collected by magnet then washed five times with wash buffer (50mM Na-phosphate pH 8.0, 300mM NaCl, 0.01% Tween-20). After the final wash, the buffer was aspirated and the Dynabeads were incubated with 100μL elution buffer (300mM imidazole, 50mM Na-phosphate pH 8.0, 300mM NaCl, 0.01% Tween-20) for 20 minutes and collected via magnet. Supernatant was combined with 25μL of 5x SDS-PAGE sample buffer and denatured for five minutes at 95 °C. 20 μL of sample was analyzed by SDS-PAGE and analyzed in a Western blot with Tetra-HIS antibody (Qiagen). Each experiment was performed in biological triplicate.

### 2.2 Trypsin digestion of samples from SDS-PAGE gel plugs

The gel plugs for each sample to be analyzed were excised by sterile razor blade, divided into 2 sections ∼1cm each, and chopped into ∼1 mm^3^ pieces. Each section was washed in dH_2_O and destained using 100 mM NH_4_HCO_3_ pH 7.5 in 50% acetonitrile. A reduction step was performed by addition of 100 μl 50 mM NH_4_HCO_3_ pH 7.5 and 10 μl of 200 mM tris(2-carboxyethyl)phosphine HCl at 37 °C for 30 min. The proteins were alkylated by addition of 100 μl of 50 mM iodoacetamide prepared fresh in 50 mM NH_4_HCO_3_ pH 7.5 buffer, and allowed to react in the dark at 20 °C for 30 min. Gel sections were washed in water, then acetonitrile, and vacuum dried. Trypsin digestion was carried out overnight at 37 °C with 1:50–1:100 enzyme–protein ratio of sequencing grade-modified trypsin (Promega) in 50 mM NH_4_HCO_3_ pH 7.5, and 20 mM CaCl_2_. Peptides were extracted with 5% formic acid and vacuum dried and sent to the Mayo Clinic Proteomics Core facility for HPLC and LC-MS/MS data acquisition.

### 2.3 HPLC for mass spectrometry

All samples were re-suspended in Burdick & Jackson HPLC-grade water containing 0.2% formic acid (Fluka), 0.1% TFA (Pierce), and 0.002% Zwittergent 3–16 (Calbiochem), a sulfobetaine detergent that contributes the following distinct peaks at the end of chromatograms: MH^+^ at 392, and in-source dimer [2 M + H^+^] at 783, and some minor impurities of Zwittergent 3–12 seen as MH^+^ at 336. The peptide samples were loaded to a 0.25 μl C_8_ OptiPak trapping cartridge custom-packed with Michrom Magic (Optimize Technologies) C8, washed, then switched in-line with a 20 cm by 75 μm C_18_ packed spray tip nano column packed with Michrom Magic C18AQ, for a 2-step gradient. Mobile phase A was water/acetonitrile/formic acid (98/2/0.2) and mobile phase B was acetonitrile/isopropanol/water/formic acid (80/10/10/0.2). Using a flow rate of 350 nl/min, a 90 min, 2-step LC gradient was run from 5% B to 50% B in 60 min, followed by 50%–95% B over the next 10 min, hold 10 min at 95% B, back to starting conditions and re-equilibrated.

### 2.4 LC-MS/MS analysis

Electrospray tandem mass spectrometry (LC–MS/MS) was performed at the Mayo Clinic Proteomics Core on a Thermo Q-Exactive Orbitrap mass spectrometer, using a 70,000 RP survey scan in profile mode, *m*/*z* 340–2000 Da, with lockmasses, followed by 20 MSMS HCD fragmentation scans at 17,500 resolution on doubly and triply charged precursors. Single charged ions were excluded, and ions selected for MS/MS were placed on an exclusion list for 60 s.

### 2.5 LC–MS/MS data analysis, statistical analysis

All LC-MS/MS *.raw Data files were analyzed with MaxQuant version 1.5.2.8, searching against the SPROT *Saccharomyces cerevisiae* database downloaded 1/9/2018, and modified by the removal of Sc HSP70 proteins Ssa1, Ssa2, Ssa3, Ssa4 and with the addition of the *Nematostella vectensis* HSP70 bait proteins (NvHsp70A, NvHsp70B, and NvHsp70D), and searched using the following criteria: LFQ quantification with a min of 1 high confidence peptide. Trypsin was selected as the protease with max miss cleavage set to 2.

Carbamidomethyl (C) was selected as a fixed modification. Variable modifications were set to Deamidation (NQ), Oxidization (M), Formylation (n-term), and Phosphorylation (STY). Orbitrap mass spectrometer was selected using a MS error of 20 ppm and a MS/MS error of 0.5 Da. A 1% FDR cutoff was selected for peptide, protein, and site identifications.

LFQ Intensities were reported based on the MS level peak areas determined by MaxQuant and reported in the proteinGroups.txt file as. Proteins were removed from this results file if they were flagged by MaxQuant as “Contaminants”, “Reverse” or “Only identified by site”. Complete three biological replicates were performed. The abundance data from each biological replicate were normalized to the ratio of the *N. vectensis* HSP70 Isoform bait protein in that run (e.g. normalized to the NvHSP70A, NvHSP70B, or NvHSP70D, respectively). LFQ Peak intensities were analyzed in each run to determine protein hits that fell into the category of either Unstressed only hits or Stressed only hits and retained if they confirmed to this state across all 3 runs. LFQ Sig cutoffs are Sig Up > 1.2 ratio (Log2 0.26) and Sig Down < 0.8 ratio (Log2 -0.32).

Any hits that were not observed in at least 2 replicates each were labeled ‘no quant’ (a normalized ratio was still calculated but not included in final data set analysis).

A list of proteins identified and corresponding ratios can be found in Supplemental Table T1. The mass spectrometry proteomic data have been deposited to the ProteomeXchange Consortium (http://proteomecentral.proteomexchange.org) [4] via the PRIDE partner repository [5,6] with the dataset identifier PXD012144.

### 2.6 Data analysis, statistical analysis and visualization

Venn diagrams were performed with Venny 2.1. Gene Ontology analysis was performed using GO Slim Mapper on the *Saccharomyces* Genome Database (http://www.yeastgenome.org/cgi-bin/GO/goSlimMapper.pl). Interactome visualization was performed using Prism 7 graphing software for individual interactomes and comparative interactomes. Data points indicating an interaction change of greater or less than a two-fold change, calculated by an average log_2_ ratio of >1 or < −1, were considered significant. In order to determine common interactors among the NvHsp70 isoforms, comparative interactomes were plotted to demonstrate binding activity of interactors between the Hsp70 isoforms. The interaction changes for each isoform are represented by a two-log ratio. The associations and dissociations between isoforms and their suggested clients in *Saccharomyces cerevisiae* with >1 or < -1 were clustered using the String Database Version 10.5.
